# The Evolution of Post-Vaccine G8P[4] Group a Rotavirus Strains in Rwanda; Notable Variance at the Neutralization Epitope Sites

**DOI:** 10.3390/pathogens12050658

**Published:** 2023-04-28

**Authors:** Peter N. Mwangi, Robyn-Lee Potgieter, Jeannine Uwimana, Leon Mutesa, Narcisse Muganga, Didier Murenzi, Lisine Tusiyenge, Jason M. Mwenda, Milton T. Mogotsi, Kebareng Rakau, Mathew D. Esona, A. Duncan Steele, Mapaseka L. Seheri, Martin M. Nyaga

**Affiliations:** 1 Next Generation Sequencing Unit, Division of Virology, Faculty of Health Sciences, University of the Free State, Bloemfontein 9300, South Africa; 2 Kigali University Teaching Hospital, College of Medicine and Health Sciences, University of Rwanda, Kigali P.O. Box 4285, Rwanda; 3 Centre for Human Genetics, College of Medicine and Health Sciences, University of Rwanda, Kigali P.O. Box 4285, Rwanda; 4 World Health Organization, Regional Office for Africa, Brazzaville P.O. Box 06, Congo; 5 Diarrhoeal Pathogens Research Unit, Sefako Makgatho Health Sciences University (MEDUNSA), Pretoria 0204, South Africa

**Keywords:** G8P[4], rotavirus, Rwanda, whole-genome sequencing, rotavirus reassortment

## Abstract

Africa has a high level of genetic diversity of rotavirus strains, which is suggested to be a possible reason contributing to the suboptimal effectiveness of rotavirus vaccines in this region. One strain that contributes to this rotavirus diversity in Africa is the G8P[4]. This study aimed to elucidate the entire genome and evolution of Rwandan G8P[4] strains. Illumina sequencing was performed for twenty-one Rwandan G8P[4] rotavirus strains. Twenty of the Rwandan G8P[4] strains had a pure DS-1-like genotype constellation, and one strain had a reassortant genotype constellation. Notable radical amino acid differences were observed at the neutralization sites when compared with cognate regions in vaccine strains potentially playing a role in neutralization escape. Phylogenetic analysis revealed that the closest relationship was with East African human group A rotavirus (RVA) strains for five of the genome segments. Two genome sequences of the NSP4 genome segment were closely related to bovine members of the DS-1-like family. Fourteen VP1 and eleven VP3 sequences had the closest relationships with the RotaTeq™ vaccine WC3 bovine genes. These findings suggest that the evolution of VP1 and VP3 might have resulted from reassortment events with RotaTeq™ vaccine WC3 bovine genes. The close phylogenetic relationship with East African G8P[4] strains from Kenya and Uganda suggests co-circulation in these countries. These findings highlight the need for continued whole-genomic surveillance to elucidate the evolution of G8P[4] strains, especially after the introduction of rotavirus vaccination.

## 1. Introduction

Human group A rotaviruses (RVA) are the leading cause of acute gastroenteritis in children globally, except in the Americas, according to 2017–2018 estimates by the Global Pediatric Diarrhea Surveillance (GPDS) network [1]. Vaccines are effective in preventing severe RVA diarrhea in young children, and the World Health Organization (WHO) has prequalified six RVA vaccines: Rotarix^®^ (GlaxoSmithKline, Rixensart, Belgium), RotaTeq^®^ (Merck & Co., Whitehouse Station, NJ, USA), Rotavac^®^ (Bharat Biotech, New Delhi, India), Rotavac 5D^®^ (Bharat Biotech, New Delhi, India), Rotasiil^®^ (Serum Institute of India Pvt Ltd., Pune, India), and Rotasiil-Liquid (Serum Institute of India Pvt Ltd., Pune, India) (https://extranet.who.int/gavi/PQ_Web/ (accessed 31 March 2023)). The Rwandan National Immunization Program began using RotaTeq^®^ in May 2012, and later switched to Rotarix^®^ in April 2017; the vaccines had a coverage rate of 89% as of 2021 (https://immunizationdata.who.int/pages/profiles/rwa.html (accessed 20 February 2023)). There was a substantial decrease of 25–44% in hospitalizations for rotavirus-induced diarrhea among children under five years old within the first three years after the introduction of the RotaTeq^®^ vaccine [2]. However, sub-optimal RVA vaccine effectiveness remains a concern, not only in Rwanda, but in the entire African continent [3].

Rotavirus is a dsRNA virus, classified within the *Sedoreoviridae* family (https://ictv.global/report/chapter/reovirales (accessed 12 February 2023)), and comprises 11 genome segments [4]. The classification of the virus has traditionally been based on the viral protein 7 (VP7) and viral protein 4 (VP4), which are designated as G and P, due to their glycoprotein nature and protease sensitivity, respectively [5]. However, the comprehensive characterization of circulating RVA strains requires a whole-genome-based approach that considers the genotype of all 11 genome segments [6]. Based on their entire genetic make-up, RVAs are further classified into two major genogroups: genogroup one, called Wa-like constellation, and genogroup, two, called DS-1-like constellation. There is also a less common third genogroup, called AU-1-like constellation [7]. The RVA whole-genome classification nomenclature is denoted as Gx-P[x]-Ix-Rx-Cx-Mx-Ax-Nx-Tx-Ex-Hx, representing the 11 genome segments: VP7, VP4, VP6, VP1, VP2, VP3, NSP1, NSP2, NSP3, NSP4, and NSP5, respectively. Each letter describes a specific property of each genome segment, and x corresponds to the number of relevant genotypes [6].

The G1, G3, G4, G9, and G12 genotypes, in combination with the P[8] genotype, are commonly linked with the Wa-like genogroup [7]. However, there have been reports of atypical strains among the G1P[8], G3P[8], and G9P[8] strains, bearing a DS-1-like genome constellation [8,9,10,11]. In contrast, the G2P[4] strains are typically linked with the DS-1-like genogroup [7]. The structure of the rotavirus genome is composed of segments that enable reassortment [4], which has contributed to the high diversity of rotavirus strains [12,13,14,15]. The vast diversity of RVA strains in Africa is attributed to one of the factors which causes the sub-optimal RVA vaccine effectiveness in the region. One of the strains contributing to this unusual diversity in Africa is the G8 genotype, which was initially only found in cattle and has been linked to zoonotic transmission in humans [16,17,18,19,20]. While the G8 genotype is uncommon globally, it has been frequently found in Africa [21,22,23,24,25,26]. Research indicates that the transmission of rotaviruses between humans and cattle may be the reason for the high incidence of G8 genotype in Africa [27,28,29].

Although rotavirus vaccines provide appreciable cross-protection against heterologous rotavirus strains [30], mathematical models indicate that even small variations in their effectiveness against certain rotavirus strains [31,32,33,34] could significantly impact the dynamics of rotavirus diseases over long periods of time [35,36,37]. Therefore, genomic surveillance of RVA strains is crucial to determine whether the major G and P antigens circulating in African regions such as Rwanda may influence the positive impact of the vaccination program. Despite this need for genomic surveillance, there is currently a dearth of data on whole genomes of human RVA circulating in Rwanda. To address this gap, we characterized the complete genomes of 21 Rwandan G8P[4] RVA strains.

## 2. Materials and Methods

### 2.1. Sample Collection

As part of an ongoing whole-genome rotavirus surveillance program conducted by the World Health Organization’s Regional Office for Africa (WHO-AFRO) in Rwanda, 158 fecal specimens were collected from children under five years old who were experiencing acute gastroenteritis. The samples, which were collected between 2011 and 2016, were then shipped to the Regional Rotavirus Reference Laboratory at the Diarrheal Pathogens Research Unit (DPRU), situated in Pretoria, South Africa, for G/P typing. Of the 158 samples, 21 were conventionally genotyped as G8P[4] and, thus, analyzed in this study.

### 2.2. Double-Stranded RNA Extraction and cDNA Synthesis

Rotavirus dsRNA extraction and cDNA synthesis were performed as described previously [14]. Briefly, the extracted RNA was incubated with 8 M lithium chloride (Sigma-Aldrich^®^, St Louis, MO, USA) for 16 h at 4 °C to enrich it for rotavirus dsRNA, then purified using a MinElute PCR purification kit (Qiagen, Hilden, Germany). Finally, electrophoresis was performed on a 5 µL aliquot of dsRNA in 1% 0.5 TBE agarose (Bioline, London, UK) gel stained with Pronasafe (Condalab, Madrid, Spain) at 95 volts for 1 h to assess the integrity of the extracted and purified rotavirus dsRNA. The cDNA synthesis was performed using the Maxima H Minus Double-Stranded cDNA Synthesis kit and protocol (ThermoFisher Scientific, Waltham, MA, USA), albeit with a slight modification of the first strand synthesis step, whereby we increased the incubation period from 30 min to 2 h. The cDNA was then purified using the MSB*®* Spin PCRapace kit (Stratec Molecular, Berlin, Germany) following the manufacturer’s instructions.

### 2.3. Preparation of DNA Library for Whole Genome Sequencing

The construction of DNA libraries was carried out in accordance with the Nextera XT DNA Library Preparation Kit and Protocol (Illumina, San Diego, CA, USA). Briefly, the process involved fragmentation of genomic DNA through tagmentation, amplification of the tagmented DNA, and purification of the post-tagmented DNA. A volume of 10 μL Tagment DNA buffer was added to each well of a PCR plate containing a 5 μL volume of purified cDNA, within the 0.2–0.3 ng/μL concentration range, followed by the addition of 5 μL of Amplicon Tagment Mix. The reaction mixture was subjected to centrifugation for 1 min at a force of 280× *g*, utilizing ambient temperature conditions. The PCR plate was placed in a thermocycler, which was programmed with a pre-heated lid option at 55 °C for 5 min, with a 10 °C hold temperature. A 5 μL volume of Neutralize Tagment buffer was added to neutralize the activity of the transposase enzyme. After centrifugation at 280× *g* and room temperature for 1 min, the PCR plate was subsequently incubated at room temperature for 5 min.

A 10 μL volume of index 1 and 2 primers was added to each sample well containing the tagmented DNA, based on the unique combinations provided on the Illumina Experimental Manager software v.1.18.1 sample sheet (Illumina Experimental Manager (IEM) Software Downloads). A 15 μL volume of Nextera PCR Mastermix was added to each well; then, the plate was centrifuged at 280× *g* at 20 °C for 1 min. The PCR program was performed on a thermocycler programmed with a pre-heated lid option at 72 °C for 3 min; for 12 cycles at 95 °C for 10 s, 55 °C for 30 s, and 72 °C for 30 s; then, finally, at 72 °C for 5 min, with a hold temperature of 10 °C.

The amplified and post-tagmented DNA library was subjected to purification, during which fragment size selection and elimination of undesired PCR contaminants was performed using AMPure XP beads (Beckman Coulter, Pasadena, CA, USA) and freshly prepared 80% ethanol. The DNA library was then validated using an Agilent 2100 BioAnalyzer (Agilent Technologies, Waldbronn, Germany). The normalized DNA libraries were combined into a single tube and denatured using sodium hydroxide. They were then diluted with hybridization buffer, and a PhiX control was added until an ultimate concentration of 8 pM was reached. Then, sequencing was performed on a MiSeq platform (Illumina, San Diego, CA, USA) for 600 paired-end cycles at the University of Free State - Next Generation Sequencing (UFS-NGS) Unit, Bloemfontein, South Africa.

### 2.4. Genome Assembly

The raw data from the sequencing process were evaluated for quality using FASTQC, version 0.11.9 [38]. Adapter sequences were eliminated using the BBDuk trimmer tool (https://sourceforge.net/projects/bbmap/ (accessed 12 January 2023)). De novo and reference-based mapping using the prototype DS-1-like reference strain (with accession numbers HQ650116-HQ650126) was performed on Geneious Prime 2023.1 [39]. A consensus was generated from the mapped data by utilizing the Geneious Consensus Tool [39].

### 2.5. Genome Genotyping and GenBanK Accession Numbers

The complete genome constellation was determined by analyzing each genome segment using the rvaGenotyper, accessible at (https://legacy.viprbrc.org/brc/rvaGenotyper.spg?method=ShowCleanInputPage&decorator=reo (accessed 17 January 2023)), in the Virus Pathogen Database and Analysis Resource (ViPR) [40]. Briefly, the user inputted the nucleotide sequence of a given rotavirus genome segment into the FASTA format. The rvaGenotyper tool then compared the sequence to a reference database of known rotavirus sequences and used a combination of nucleotide alignments and genotype-specific cut-off values to determine the genotype of the input sequence. The accession numbers assigned to all gene sequences in this study were OQ201345-OQ201575, and these were deposited into the NCBI GenBank database (Supplementary Table S1).

### 2.6. Sequence and Phylogenetic Analyses

The MUSCLE tool [41] in the MEGA 11 software [42] was utilized for comparative analysis of the ORFs. The DNA Model Test program in MEGA 11 was used to determine the most suitable evolutionary model. Phylogenetic trees, using maximum likelihood (ML), were generated for each genome segment, and branch support was evaluated through 1000 bootstrap replicates. In order to determine the genetic similarities between strains for each gene, the p-distance algorithm, which is available in MEGA 11, was employed. The Virus Variation Resource of the National Center for Biotechnology Information (NCBI) and the basic local alignment search tool (BLAST) were employed to compile reference sequences [43,44]. The accession numbers of these reference sequences, which were used in the construction of the ML trees, are included in the Supplementary Table S2.

## 3. Results

### 3.1. Full Genotyping Results

The complete sequences of all 11 genomic segments of 21 Rwandan G8P[4] strains were determined (Supplementary Table S3). Twenty of the Rwandan G8P[4] strains were assigned to the DS-1-like genotype constellation (G8-P[4]-I2-R2-C2-M2-A2-N2-T2-E2-H2), while one strain had a Wa-like NSP2 genotype and bore the reassortant genomic constellation (G8-P[4]-I2-R2-C2-M2-A2-N1-T2-E2-H2) (Table 1).

**Table 1:** Rwandan G8P[4] strains and their genotype constellations.

Rwandan G8P[4] Strains	Genotype Constellations										
	VP7	VP4	VP6	VP1	VP2	VP3	NSP1	NSP2	NSP3	NSP4	NSP5
RVA/Human-wt/RWA/UFS-NGS-MRC-DPRU441/2012/G8P[4]	G8 **(V)**	P[4] **(II)**	I2 **(V)**	R2 **(V)**	C2 **(IV)**	M2 **(V)**	A2 **(IV)**	N2 **(V)**	T2 **(V)**	E2 **(XXIII)**	H2 **(IV)**
RVA/Human-wt/RWA/UFS-NGS-MRC-DPRU478/2013/G8P[4]	G8 **(V)**	P[4] **(II)**	I2 **(V)**	R2 **(XII)**	C2 **(IV)**	M2 **(X)**	A2 **(IV)**	N1	T2 **(V)**	E2 **(XXIII)**	H2 **(IV)**
RVA/Human-wt/RWA/UFS-NGS-MRC-DPRU582/2013/G8P[4]	G8 **(V)**	P[4] **(II)**	I2 **(V)**	R2 **(V)**	C2 **(IV)**	M2 **(V)**	A2 **(IV)**	N2 **(V)**	T2 **(V)**	E2 **(XXIII)**	H2 **(IV)**
RVA/Human-wt/RWA/UFS-NGS-MRC-DPRU589/2013/G8P[4]	G8 **(V)**	P[4] **(II)**	I2 **(V)**	R2 **(V)**	C2 **(IV)**	M2 **(V)**	A2 **(IV)**	N2 **(V)**	T2 **(V)**	E2 **(XXIII)**	H2 **(IV)**
RVA/Human-wt/RWA/UFS-NGS-MRC-DPRU590/2013/G8P[4]	G8 **(V)**	P[4] **(II)**	I2 **(V)**	R2 **(XII)**	C2 **(XIII)**	M2 **(V)**	A2 **(IV)**	N2 **(V)**	T2 **(V)**	E2 **(XV)**	H2 **(IV)**
RVA/Human-wt/RWA/UFS-NGS-MRC-DPRU596/2013/G8P[4]	G8 **(V)**	P[4] **(II)**	I2 **(V**	R2 **(V)**	C2 **(IV)**	M2 **(V)**	A2 **(IV)**	N2 **(V)**	T2 **(V)**	E2 **(XXIII)**	H2 **(IV)**
RVA/Human-wt/RWA/UFS-NGS-MRC-DPRU599/2013/G8P[4]	G8 **(V)**	P[4] **(II)**	I2 **(V)**	R2 **(XII)**	C2 **(IV)**	M2 **(X)**	A2 **(IV)**	N2 **(V)**	T2 **(V)**	E2 **(XXIII)**	H2 **(IV)**
RVA/Human-wt/RWA/UFS-NGS-MRC-DPRU602/2013/G8P[4]	G8 **(V)**	P[4] **(II)**	I2 **(V)**	R2 **(XII)**	C2 **(IV)**	M2 **(X)**	A2 **(IV)**	N2 **(V)**	T2 **(V)**	E2 **(XXIII)**	H2 **(IV)**
RVA/Human-wt/RWA/UFS-NGS-MRC-DPRU607/2013/G8P[4]	G8 **(V)**	P[4] **(II)**	I2 **(V)**	R2 **(XII)**	C2 **(IV)**	M2 **(X)**	A2 **(IV)**	N2 **(V)**	T2 **(V)**	E2 **(XXIII)**	H2 **(IV)**
RVA/Human-wt/RWA/UFS-NGS-MRC-DPRU620/2013/G8P[4]	G8 **(V)**	P[4] **(II)**	I2 **(V)**	R2 **(XII)**	C2 **(IV)**	M2 **(X)**	A2 **(IV)**	N2 **(V)**	T2 **(V)**	E2 **(XXIII)**	H2 **(IV)**
RVA/Human-wt/RWA/UFS-NGS-MRC-DPRU632/2013/G8P[4]	G8 **(V)**	P[4] **(II)**	I2 **(V)**	R2 **(XII)**	C2 **(IV)**	M2 **(X)**	A2 **(IV)**	N2 **(V)**	T2 **(V)**	E2 **(XXIII)**	H2 **(IV)**
RVA/Human-wt/RWA/UFS-NGS-MRC-DPRU637/2013/G8P[4]	G8 **(V)**	P[4] **(II)**	I2 **(V)**	R2 **(XII)**	C2 **(IV)**	M2 **(X)**	A2 **(IV)**	N2 **(V)**	T2 **(V)**	E2 **(XXIII)**	H2 **(IV)**
RVA/Human-wt/RWA/UFS-NGS-MRC-DPRU642/2013/G8P[4]	G8 **(V)**	P[4] **(II)**	I2 **(V)**	R2 **(V)**	C2 **(IV)**	M2 **(V)**	A2 **(IV)**	N2 **(V)**	T2 **(V)**	E2 **(XXIII)**	H2 **(IV)**
RVA/Human-wt/RWA/UFS-NGS-MRC-DPRU652/2013/G8P[4]	G8 **(V)**	P[4] **(II)**	I2 **(V)**	R2 **(XII)**	C2 **(IV)**	M2 **(X)**	A2 **(IV)**	N2 **(V)**	T2 **(V)**	E2 **(XXIII)**	H2 **(IV)**
RVA/Human-wt/RWA/UFS-NGS-MRC-DPRU653/2013/G8P[4]	G8 **(V)**	P[4] **(II)**	I2 **(V**	R2 **(V)**	C2 **(IV)**	M2 **(V)**	A2 **(IV)**	N2 **(V)**	T2 **(V)**	E2 **(XXIII)**	H2 **(IV)**
RVA/Human-wt/RWA/UFS-NGS-MRC-DPRU656/2013/G8P[4]	G8 **(V)**	P[4] **(II)**	I2 **(V)**	R2 **(XII**	C2 **(XIII)**	M2 **(V)**	A2 **(IV)**	N2 **(V)**	T2 **(V)**	E2 **(XV)**	H2 **(IV)**
RVA/Human-wt/RWA/UFS-NGS-MRC-DPRU661/2013/G8P[4]	G8 **(V)**	P[4] **(II)**	I2 **(V)**	R2 **(XII)**	C2 **(IV)**	M2 **(V)**	A2 **(IV)**	N2 **(V)**	T2 **(V)**	E2 **(XXIII)**	H2 **(IV)**
RVA/Human-wt/RWA/UFS-NGS-MRC-DPRU666/2013/G8P[4]	G8 **(V)**	P[4] **(II)**	I2 **(V)**	R2 **(XII)**	C2 **(IV)**	M2 **(X)**	A2 **(IV)**	N2 **(V)**	T2 **(V)**	E2 **(XXIII)**	H2 **(IV)**
RVA/Human-wt/RWA/UFS-NGS-MRC-DPRU667/2013/G8P[4]	G8 **(V)**	P[4] **(II)**	I2 **(V)**	R2 **(XII)**	C2 **(IV)**	M2 **(X)**	A2 **(IV)**	N2 **(V)**	T2 **(V)**	E2 **(XXIII)**	H2 **(IV)**
RVA/Human-wt/RWA/UFS-NGS-MRC-DPRU737/2013/G8P[4]	G8 **(V)**	P[4] **(II)**	I2 **(V)**	R2 **(XII)**	C2 **(IV)**	M2 **(X)**	A2 **(IV)**	N2 **(V)**	T2 **(V)**	E2 **(XXIII)**	H2 **(IV)**
RVA/Human-wt/RWA/UFS-NGS-MRC-DPRU7997/2015/G8P[4]	G8 **(V)**	P[4] **(II)**	I2 **(V)**	R2 **(V)**	C2 **(IV)**	M2 **(V)**	A2 **(IV)**	N2 **(V)**	T2 **(V)**	E2 **(XXIII)**	H2 **(IV)**

**Figure 1:**
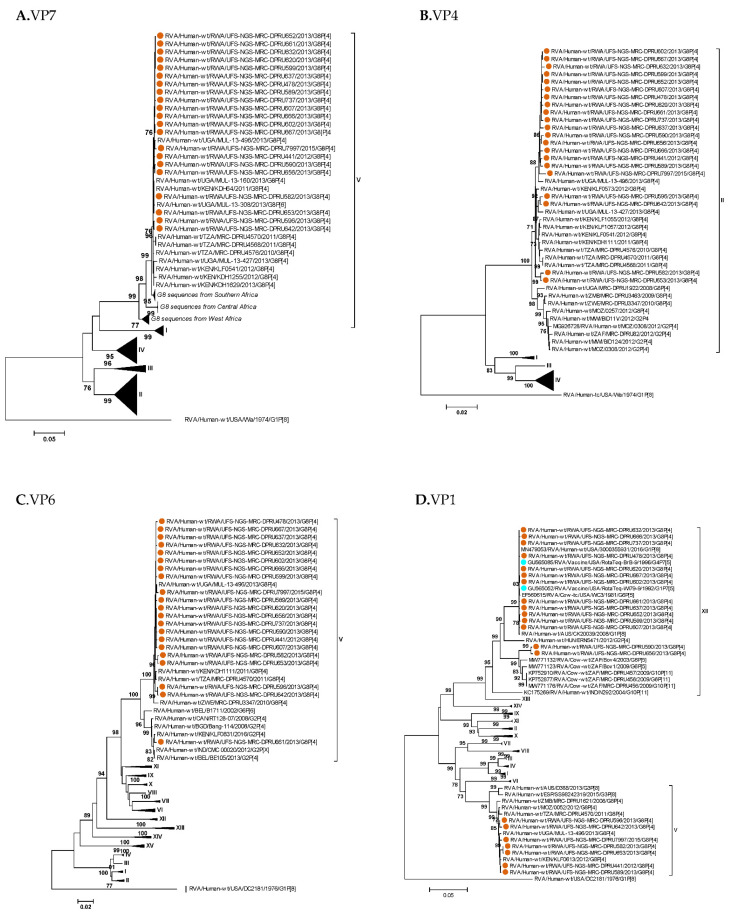

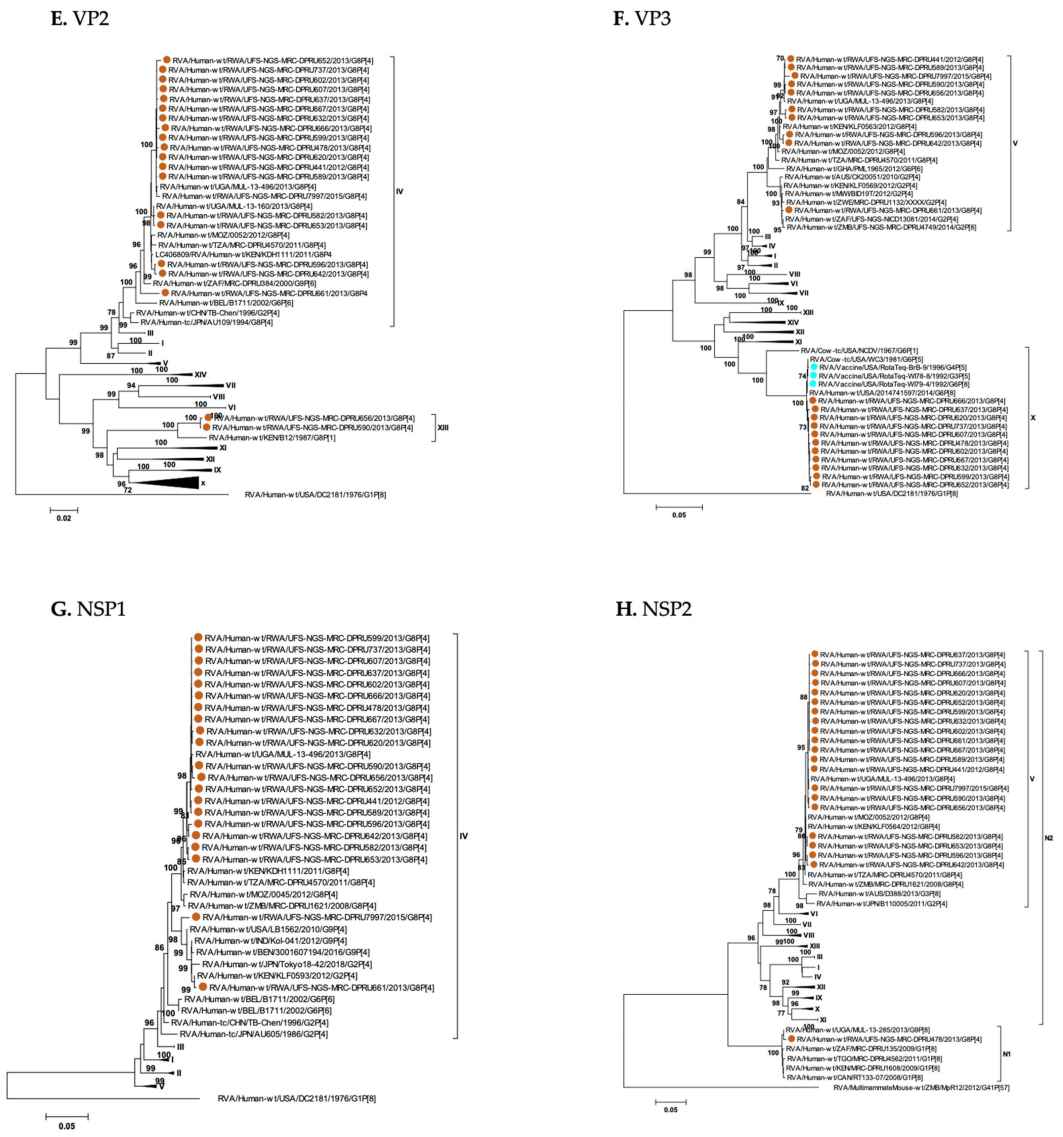

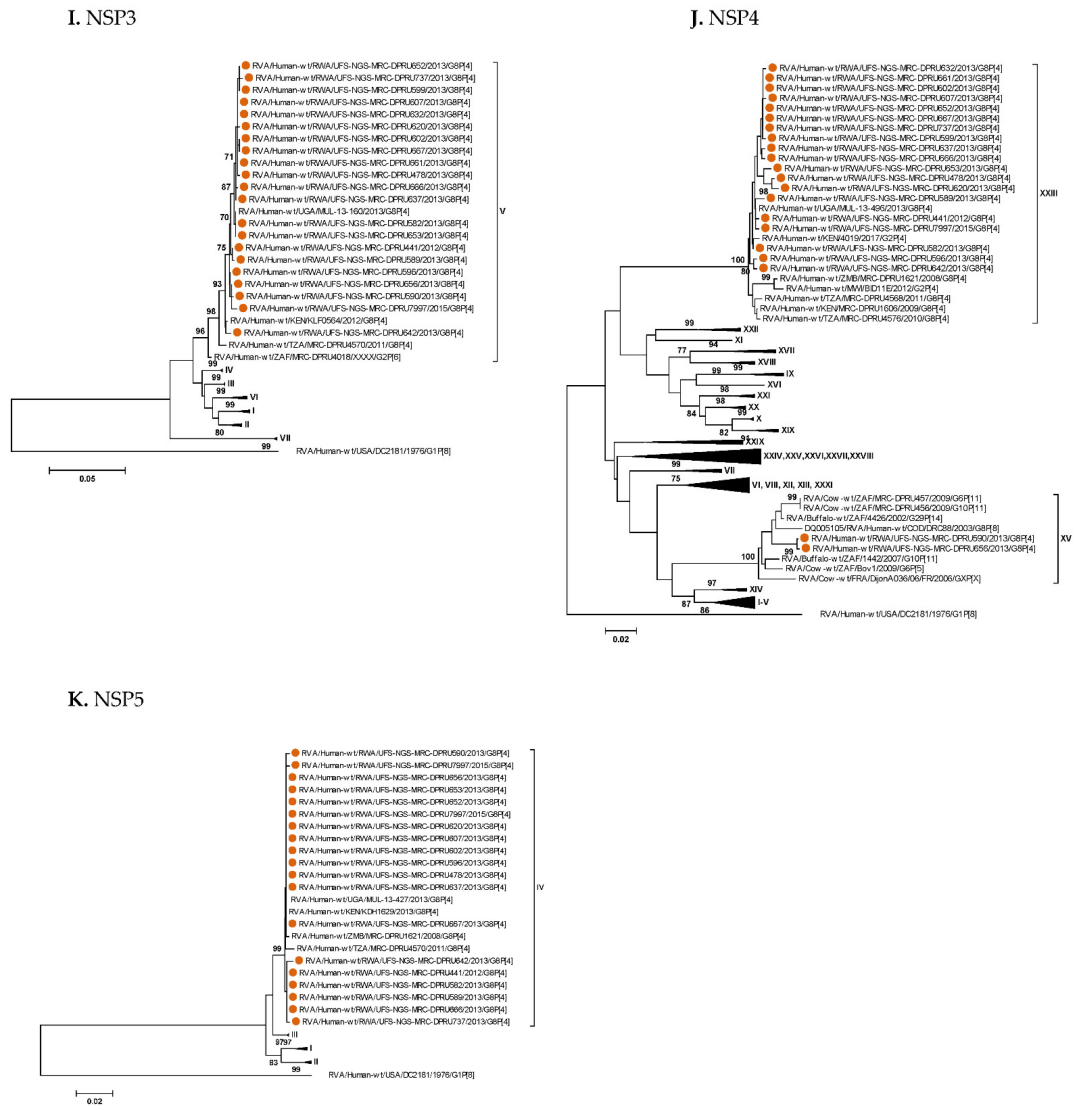
Maximum likelihood phylogenetic trees of the genome segment encoding (**A**) VP7, (**B**) VP4, (**C**) VP6, (**D**) VP1, (**E**) VP2, (**F**) VP3, (**G**) NSP1, (**H**) NSP2, (**I**) NSP3, (**J**) NSP4, and (**K**) NSP5. The brown-red circular symbols represent the study strains. The lineages are indicated using Roman numerals. The scale number indicates the number of nucleotide substitutions per site.

Color-coding represents genogroup assignment. The light blue color is linked with the G8 genotype; the light red color is associated with the DS-1-like genogroup; and the light green color is associated with the Wa-like genogroup. The nomenclature of the rotavirus strains is based on the Rotavirus Classification Working Group (https://rega.kuleuven.be/cev/viralmetagenomics/virus-classification/rcwg (accessed 15 January 2023)). Roman numerals in parentheses after the genotype names indicate the lineage, as observed in the phylogenetic trees in Figure 1A–K.

### 3.2. Analysis of Neutralization Epitopes

A comparison of the amino acids in the neutralization epitopes between Rwandan G8 VP7 genes and cognate VP7 genes of the vaccine strains [45] showed that only five of the 29 amino acid residues were fully conserved among all the Rwandan G8 strains (Table 2). The Rwandan G8 strains displayed 24 amino acid differences from the vaccine strains’ VP7 component (Table 2). Apart from N91T, V/M129I, and R143K, the rest of the amino acid differences involved a change in either charge or polarity [46].

The neutralization epitopes of the Rwandan VP4 gene shared 27 identical amino acid residues with the cognate residues in the VP4 component of RotaTeq™ and Rotarix*®* vaccines [47] (Table 3). The observed amino acid differences were mostly found in the 8-1 and 8-3 epitopes (Table 3). The VP4 antigenic component of Rwandan G8P[4] strains showed seven amino acid differences (N192D, N113S, P114Q, V115T, D116N, R/S131E, and D133N) in comparison to the cognate regions in RotaTeq™ and Rotarix^®^ (Table 3). Except for the change in the N113S amino acid, the rest of the amino acid differences were considered to be radical changes, as they involved altering either the charge or the polarity of the amino acid [46].

### 3.3. Phylogenetic Analyses

To determine the genetic relationship between Rwandan G8P[4] strains and other RVA strains from GenBank, we conducted phylogenetic analyses based on the nucleotide sequences of the complete ORFs of all 11 genome segments. We used previously described lineage framework for the G8 VP7 genes [48] and the P[4] genes [49]. The lineage framework for the G8 VP7 genes was based on maximum likelihood phylogenetic analysis of 246 G8 VP7 genes, which identified five G8 lineages [48], while for P[4] VP4 genes, it was based on global P[4] phylogenetic analysis [49]. We also utilized a lineage framework that categorized the nine genotype two backbone genes [50]. This lineage framework defined lineages for genes of genotype two based on stringent bootstrap support and pairwise analysis of the nucleotide sequences [50]. Selected reference sequences for each lineage were utilized in this study to determine the lineage of a particular genotype two strain.

The Rwandan G8 study sequences and GenBank G8 sequences (*n* = 180) segregated into five G8 lineages (Figure 1A). Rwandan G8 sequences clustered lineage V into a sub-lineage comprising sequences predominantly from East Africa, and the highest nucleotide similarities (99.7–100%) were observed with a Kenyan G8 genotype (Table 4; Figure 1A).

The P[4] sequences from the Rwandan study and the 43 selected GenBank P[4] reference sequences segregated into four P[4] lineages, and all fell under lineage II, which mainly consisted of African P[4] sequences (Figure 1B). The VP4 genes from Rwandan G8P[4] strains showed the highest nucleotide similarities (ranging from 98.8% to 100%) with a Kenyan strain (Table 4; Figure 1B). The Rwandan VP6 sequences clustered in lineage V (Figure 1C), and within this lineage, the highest nucleotide similarities, ranging from 99.5% to 99.9%, were observed with a Ugandan strain (Table 4; Figure 1C). Rwandan R2 sequences segregated into two lineages, lineage V and lineage XII (Figure 1D), which were distantly related to each other, with nucleotide identities ranging from 85.2–100% (Supplementary Table S4). A total of seven Rwandan G8P[4] VP1 sequences clustered with other human R2 gene sequences, mainly from Africa, in lineage V (Figure 1D), and the highest nucleotide similarities (99.6–99.9%) were observed with a Kenyan strain (Table 4; Figure 1D). The remaining fourteen Rwandan R2 sequences clustered in lineage XII, a bovine lineage consisting of cow sequences, including the RotaTeq™ vaccine bovine genes (Figure 1D). In this lineage, the highest nucleotide homology (99.9–100%) was observed with the RotaTeq™ vaccine strain (Table 4). In a different sub-lineage within lineage XII, two Rwandan R2 sequences showed the highest nucleotide identity (97.5–97.6%) with a South African bovine strain (Table 4; Figure 1D). The VP2 genes of Rwandan G8P[4] strains showed a distant relationship with each other, ranging from 85.7–100% (Supplementary Table S4), and clustered separately into two lineages within the C2 genotype (Figure 1E). Nineteen VP2 genes clustered together with other human C2 genes in lineage IV (Figure 1E), with highest nucleotide similarities to a Ugandan strain ranging from 99.5–99.9% (Table 4; Figure 1E). The other two Rwandan G8P[4] VP2 genes, clustered in lineage XIII (Figure 1E), with the highest nucleotide similarities (96.3–96.4%) found with a Kenyan G8P[1] strain (Figure 1E). The VP3 genes of the Rwandan G8P[4] strains were distantly related to one another (ranging from 82.2–100% similarity) (Supplementary Table S4). The VP3 genes formed separate clusters, with ten of them found in lineage V (Figure 1F). These VP3 genes had the highest similarity (ranging from 99.3–99.9%) to a Ugandan strain (Table 4; Figure 1F). The remaining 11 VP3 genes of the Rwandan G8P[4] strains were found in lineage X, a hybrid lineage of human and bovine M2 genes (Figure 1F). These VP3 genes had the highest level of similarity (ranging from 99.6–99.9%) with the VP3 bovine gene of a RotaTeq™ vaccine strain (Table 4; Figure 1F).

The NSP1 genes of the Rwandan G8P[4] strains clustered in lineage IV (Figure 1G), and had the highest level of nucleotide similarities (99.3–99.9%) with a Ugandan strain (Table 4; Figure 1G). The NSP2 gene sequences of Rwandan G8P[4] were segregated into two genotypes, N1 and N2 (Figure 1H). One sequence clustered into genotype N1, while the other twenty sequences clustered into genotype N2 (Figure 1H). The 20 N2 genes of Rwandan G8P[4] strains that clustered into human lineage V had the highest level of similarity (99.6–100%) to a Ugandan strain (Table 4). The one strain with the N1 genotype had the closest nucleotide similarity (99.7%) to the NSP2 gene (N1 genotype) of a G9P[8] Ugandan strain (Figure 1H). Rwandan T2 gene sequences clustered into lineage V alongside other human T2 strains, with the highest nucleotide similarities (99.4–100%) observed with a Ugandan strain (Table 4; Figure 1I). The Rwandan E2 gene sequences had a distant relationship (ranging from 82.7–100%) among themselves (Supplementary Table S4), and clustered separately into two different lineages within the E2 genotype (Figure 1J). Nineteen NSP4 genes clustered together with other human M2 genes in lineage XXIII (Figure 1J), with high nucleotide similarities, ranging from 99.2–100%, to a Ugandan strain (Table 4; Figure 1J). The remaining two Rwandan G8P[4] NSP4 genes clustered in lineage XV, a hybrid lineage comprising both human and animal E2 genes (Figure 1J), and the highest nucleotide similarities ranged from 97.5–97.7% with the NSP4 gene of a South African bovine strain (Table 4; Figure 1J). Rwandan H2 gene sequences clustered in lineage IV alongside other human H2 genes (Figure 1K), with the highest nucleotide similarities observed with a Kenyan strain (Figure 1K).

## 4. Discussion

The study provides a whole-genome analysis of the genome sequences of 21 Rwandan G8P[4] strains, revealing that 20 of the strains exhibited pure DS-1-like genotype constellations, consistent with reports from other parts of the world [21,48,51,52]. It is suggested that human RVA with pure genome constellations in the same genogroup could have co-evolved to generate sets of proteins that function optimally when maintained together [53]. Notably, one strain exhibited inter-genogroup reassortment at the NSP2 genome segment, and this reassortment phenomenon has also been reported in another G8P[4] study [54]. Reassortment of rotavirus genome segments is a relatively frequent phenomenon that generates reassortant rotavirus strains [11,12,15,55,56,57,58]. These findings underscore the genotype constellation diversity of G8P[4] strains.

The close phylogenetic relationship of the G8 and P[4] genes with contemporary RVA human strains from Kenya and Uganda suggest co-circulation in these neighboring countries. Rotaviruses are highly contagious and can spread easily between individuals and populations [59]; this finding highlights the need for genetic data to be shared between neighboring countries to track circulating RVA strains. The clustering of the backbone genome segments of Rwandan G8P[4] RVA strains showed that not all of these genes evolved in the same way. The high degree of homology and clustering of some genes with contemporary human RVA strains from Kenya and Uganda suggests local evolution, while the clustering of other genes with artiodactyl genes suggests possible interspecies reassortment.

Notably, for the VP1 and VP3 genes, we observed the closest phylogenetic relationship with RotaTeq™ vaccine WC3 bovine genes, indicating possible reassortment between wild-type DS-1-like and RotaTeq™ WC3 bovine genes. The RotaTeq™ vaccine comprises five reassortants of human–bovine (WC3) rotaviruses, each of which has a bovine/WC3 core and a surface protein derived from a human rotavirus, namely, G1, G2, G3, G4, or P[8] [60]. Reassortment between RotaTeq™ vaccine strains and wild-type strains has been reported in other studies [15,61,62,63,64]. It remains to be explored whether vaccine-derived strains could cause an increase in virulence. According to some studies, mutations occurring either de novo or through the selection of pre-existing minor variants in the vaccine may cause vaccine strains to revert to increased virulence [65,66,67,68,69].

The study found amino acid differences in the neutralization epitope regions between the VP7 study strains and the vaccine strains (Rotarix^®^, RotaTeq™, Rotavac^®,^ and Rotasiil^®^), were mostly radical, meaning they involved changes in charge or polarity [46]. Amino acid differences in positions 94, 96, 147, 148, 190, 211, 213, and 217 have been identified as significant in altering the antigenicity of rotaviruses and potentially contributing to neutralization escape [70,71]. Similarly, the amino acid differences observed between the neutralization epitopes in the VP4 study strains and vaccine strains (Rotarix^®^ and RotaTeq™) were mostly radical, and could contribute to escape of host immunity [47,72].

Although analyzing pre-vaccination Rwanda G8P[4] samples would have provided more comprehensive insights and this was not feasible due to limited availability. Furthermore, the prevalence of circulating rotavirus strains is known to naturally fluctuate [73], which further impedes in-depth analysis. Other limitations of our study include a need for more detailed demographic data for a deeper interpretation of the presented data and the fact that our samples were collected a decade ago. Despite these limitations, we believe performing a whole-genome characterization of this uncommon rotavirus strain would still be valuable. Our study provides significant insights into the evolution of G8P[4] strains in Rwanda, which could be useful in predicting their presence in neighboring regions.

## 5. Conclusions

The results demonstrated a close evolutionary relationship of Rwandan G8P[4] with other African strains, especially East African G8P[4] strains from Kenya and Uganda, an indication of their co-circulation in this region. Notable radical amino acid differences, which were observed at the neutralization sites when compared with cognate regions in vaccine strains, require further investigation, as they could potentially play a role in neutralization escape. Our findings also suggest the existence of reassortment events between co-circulating human DS-1-like RVA strains with bovine and RotaTeq™ WC3 bovine backbone genes. The high homology and phylogenetic clustering with RotaTeq™ WC3 bovine genes in the VP1 and VP3 genome segments were rather unexpected. Continued whole-genome surveillance of RVA strains is essential to evaluate the effect of RVA vaccines and to provide insights into the frequency of reassortment events that occur naturally, as well as the epidemiological fitness of RVA strains resulting from these events.

## Figures and Tables

**Table 2:** Differences in amino acid composition of neutralization epitopes between Rwandan G8 VP7 strains and the VP7 components of the rotavirus vaccine strains.

		Neutralization Epitopes																												
								7-1a										7-1b								7-2				
		87	91	94	96	97	98	99	100	104	123	125	129	130	291	201	211	212	213	238	242	143	145	146	147	148	190	217	221	264
**Vaccine strains**	**JN849114/RVA/Vaccine/USA/Rotarix-A41CB052A/1988/G1P[8]**	**T**	**T**	**N**	**G**	**E**	**W**	**K**	**D**	**Q**	**S**	**V**	**V**	**D**	**K**	**Q**	**N**	**V**	**D**	**N**	**T**	**K**	**D**	**Q**	**N**	**L**	**S**	**M**	**N**	**G**
	**GU565057/RVA/Vaccine/USA/RotaTeq-WI79-9/1992/G1P[5]**	**T**	**T**	**N**	**G**	**D**	**W**	**K**	**D**	**Q**	**S**	**V**	**V**	**D**	**K**	**Q**	**N**	**V**	**D**	**N**	**T**	**K**	**D**	**Q**	**S**	**L**	**S**	**M**	**N**	**G**
	**GU565068/RVA/Vaccine/USA/RotaTeq-SC2-9/1992/G2P[5]**	**A**	**N**	**S**	**D**	**E**	**W**	**E**	**N**	**Q**	**D**	**T**	**M**	**N**	**K**	**Q**	**D**	**V**	**S**	**N**	**S**	**R**	**D**	**N**	**T**	**S**	**D**	**I**	**S**	**G**
	**GU565079/RVA/Vaccine/USA/RotaTeq-WI78-8/1992/G3P[5]**	**T**	**T**	**N**	**N**	**S**	**W**	**K**	**D**	**Q**	**D**	**A**	**V**	**D**	**K**	**Q**	**D**	**A**	**N**	**K**	**D**	**K**	**D**	**A**	**T**	**L**	**S**	**E**	**A**	**G**
	**GU565090/RVA/Vaccine/USA/RotaTeq-BrB-9/1996/G4P[5]**	**S**	**T**	**S**	**T**	**E**	**W**	**K**	**D**	**Q**	**N**	**L**	**I**	**D**	**K**	**Q**	**D**	**T**	**A**	**D**	**T**	**R**	**A**	**S**	**G**	**E**	**S**	**T**	**S**	**G**
	**GU565046/RVA/Vaccine/USA/RotaTeq-WI79-4/1992/G6P[8]**	**V**	**N**	**A**	**T**	**E**	**W**	**K**	**D**	**Q**	**D**	**A**	**V**	**E**	**K**	**Q**	**N**	**P**	**D**	**N**	**A**	**K**	**D**	**S**	**T**	**Q**	**S**	**T**	**T**	**G**
	**FJ361209/RVA/Vaccine/IND/Rotavac-116E/AG/G9P[11]**	**I**	**T**	**G**	**T**	**E**	**W**	**K**	**G**	**Q**	**D**	**A**	**I**	**D**	**K**	**Q**	**N**	**T**	**A**	**D**	**N**	**K**	**N**	**S**	**T**	**L**	**S**	**E**	**N**	**G**
	**AB045372/RVA/Vaccine/IND/Rotasill-Au32/2016/G9**	**A**	**T**	**G**	**T**	**E**	**W**	**K**	**D**	**Q**	**D**	**A**	**I**	**D**	**K**	**Q**	**N**	**T**	**A**	**D**	**T**	**K**	**D**	**S**	**T**	**L**	**S**	**E**	**S**	**G**
**Study strains**	**RVA/Human-wt/RWA/UFS-NGS-MRC-DPRU441/2012/G8P[4]**	**A**	**T**	**A**	**S**	**S**	**W**	**K**	**D**	**Q**	**D**	**A**	**I**	**N**	**K**	**Q**	**D**	**T**	**T**	**N**	**T**	**K**	**N**	**A**	**D**	**S**	**S**	**E**	**A**	**G**
	**RVA/Human-wt/RWA/UFS-NGS-MRC-DPRU478/2013/G8P[4]**	**A**	**T**	**A**	**S**	**S**	**W**	**K**	**D**	**Q**	**D**	**A**	**I**	**N**	**K**	**Q**	**D**	**T**	**T**	**N**	**T**	**K**	**N**	**A**	**D**	**S**	**S**	**E**	**A**	**G**
	**RVA/Human-wt/RWA/UFS-NGS-MRC-DPRU582/2013/G8P[4]**	**A**	**T**	**A**	**S**	**S**	**W**	**K**	**D**	**Q**	**D**	**A**	**I**	**N**	**K**	**Q**	**D**	**T**	**T**	**N**	**T**	**K**	**N**	**A**	**D**	**S**	**S**	**E**	**A**	**G**
	**RVA/Human-wt/RWA/UFS-NGS-MRC-DPRU589/2013/G8P[4]**	**A**	**T**	**A**	**S**	**S**	**W**	**K**	**D**	**Q**	**D**	**A**	**I**	**N**	**K**	**Q**	**D**	**T**	**T**	**N**	**T**	**K**	**N**	**A**	**D**	**S**	**S**	**E**	**A**	**G**
	**RVA/Human-wt/RWA/UFS-NGS-MRC-DPRU590/2013/G8P[4]**	**A**	**T**	**A**	**S**	**S**	**W**	**K**	**D**	**Q**	**D**	**A**	**I**	**N**	**K**	**Q**	**D**	**T**	**T**	**N**	**T**	**K**	**N**	**A**	**D**	**S**	**S**	**E**	**A**	**G**
	**RVA/Human-wt/RWA/UFS-NGS-MRC-DPRU596/2013/G8P[4]**	**A**	**T**	**A**	**S**	**S**	**W**	**K**	**D**	**Q**	**D**	**A**	**I**	**N**	**K**	**Q**	**D**	**T**	**T**	**N**	**T**	**K**	**N**	**A**	**N**	**S**	**S**	**E**	**A**	**G**
	**RVA/Human-wt/RWA/UFS-NGS-MRC-DPRU599/2013/G8P[4]**	**A**	**T**	**A**	**S**	**S**	**W**	**K**	**D**	**Q**	**D**	**A**	**I**	**N**	**K**	**Q**	**D**	**T**	**T**	**N**	**T**	**K**	**N**	**A**	**D**	**S**	**S**	**E**	**A**	**G**
	**RVA/Human-wt/RWA/UFS-NGS-MRC-DPRU602/2013/G8P[4]**	**A**	**T**	**A**	**S**	**S**	**W**	**K**	**D**	**Q**	**D**	**A**	**I**	**N**	**K**	**Q**	**D**	**T**	**T**	**N**	**T**	**K**	**N**	**A**	**D**	**S**	**S**	**E**	**A**	**G**
	**RVA/Human-wt/RWA/UFS-NGS-MRC-DPRU607/2013/G8P[4]**	**A**	**T**	**A**	**S**	**S**	**W**	**K**	**D**	**Q**	**D**	**A**	**I**	**N**	**K**	**Q**	**D**	**T**	**T**	**N**	**T**	**K**	**N**	**A**	**D**	**S**	**S**	**E**	**A**	**G**
	**RVA/Human-wt/RWA/UFS-NGS-MRC-DPRU620/2013/G8P[4]**	**A**	**T**	**A**	**S**	**S**	**W**	**K**	**D**	**Q**	**D**	**A**	**I**	**N**	**K**	**Q**	**D**	**T**	**T**	**N**	**T**	**K**	**N**	**A**	**D**	**S**	**S**	**E**	**A**	**G**
	**RVA/Human-wt/RWA/UFS-NGS-MRC-DPRU632/2013/G8P[4]**	**A**	**T**	**A**	**S**	**S**	**W**	**K**	**D**	**Q**	**D**	**A**	**I**	**N**	**K**	**Q**	**D**	**T**	**T**	**N**	**T**	**K**	**N**	**A**	**D**	**S**	**S**	**E**	**A**	**G**
	**RVA/Human-wt/RWA/UFS-NGS-MRC-DPRU637/2013/G8P[4]**	**A**	**T**	**A**	**S**	**S**	**W**	**K**	**D**	**Q**	**D**	**A**	**I**	**N**	**K**	**Q**	**D**	**T**	**T**	**N**	**T**	**K**	**N**	**A**	**D**	**S**	**S**	**E**	**A**	**G**
	**RVA/Human-wt/RWA/UFS-NGS-MRC-DPRU642/2013/G8P[4]**	**A**	**T**	**A**	**S**	**S**	**W**	**K**	**D**	**Q**	**D**	**A**	**I**	**N**	**K**	**Q**	**D**	**T**	**T**	**N**	**T**	**K**	**N**	**A**	**N**	**S**	**S**	**E**	**A**	**G**
	**RVA/Human-wt/RWA/UFS-NGS-MRC-DPRU652/2013/G8P[4]**	**A**	**T**	**A**	**S**	**S**	**W**	**K**	**D**	**Q**	**D**	**A**	**I**	**N**	**K**	**Q**	**D**	**T**	**T**	**N**	**T**	**K**	**N**	**A**	**D**	**S**	**S**	**E**	**A**	**G**
	**RVA/Human-wt/RWA/UFS-NGS-MRC-DPRU653/2013/G8P[4]**	**A**	**T**	**A**	**S**	**S**	**W**	**K**	**D**	**Q**	**D**	**A**	**I**	**N**	**K**	**Q**	**D**	**T**	**T**	**N**	**T**	**K**	**N**	**A**	**D**	**S**	**S**	**E**	**A**	**G**
	**RVA/Human-wt/RWA/UFS-NGS-MRC-DPRU656/2013/G8P[4]**	**A**	**T**	**A**	**S**	**S**	**W**	**K**	**D**	**Q**	**D**	**A**	**I**	**N**	**K**	**Q**	**D**	**T**	**T**	**N**	**T**	**K**	**N**	**A**	**D**	**S**	**S**	**E**	**A**	**G**
	**RVA/Human-wt/RWA/UFS-NGS-MRC-DPRU661/2013/G8P[4]**	**A**	**T**	**A**	**S**	**S**	**W**	**K**	**D**	**Q**	**D**	**A**	**I**	**N**	**K**	**Q**	**D**	**T**	**T**	**N**	**T**	**K**	**N**	**A**	**D**	**S**	**S**	**E**	**A**	**G**
	**RVA/Human-wt/RWA/UFS-NGS-MRC-DPRU666/2013/G8P[4]**	**A**	**T**	**A**	**S**	**S**	**W**	**K**	**D**	**Q**	**D**	**A**	**I**	**N**	**K**	**Q**	**D**	**T**	**T**	**N**	**T**	**K**	**N**	**A**	**D**	**S**	**S**	**E**	**A**	**G**
	**RVA/Human-wt/RWA/UFS-NGS-MRC-DPRU667/2013/G8P[4]**	**A**	**T**	**A**	**S**	**S**	**W**	**K**	**D**	**Q**	**D**	**A**	**I**	**N**	**K**	**Q**	**D**	**T**	**T**	**N**	**T**	**K**	**N**	**A**	**D**	**S**	**S**	**E**	**A**	**G**
	**RVA/Human-wt/RWA/UFS-NGS-MRC-DPRU737/2013/G8P[4]**	**A**	**T**	**A**	**S**	**S**	**W**	**K**	**D**	**Q**	**D**	**A**	**I**	**N**	**K**	**Q**	**D**	**T**	**T**	**N**	**T**	**K**	**N**	**A**	**D**	**S**	**S**	**E**	**A**	**G**
	**RVA/Human-wt/RWA/UFS-NGS-MRC-DPRU7997/2015/G8P[4]**	**A**	**T**	**A**	**S**	**S**	**W**	**K**	**D**	**Q**	**N**	**A**	**I**	**N**	**K**	**Q**	**D**	**T**	**T**	**N**	**T**	**K**	**N**	**A**	**D**	**S**	**S**	**E**	**A**	**G**

Alignment of epitope residues in VP7 between the wild-type G8 strains and the strains present in Rotarix^®^, RotaTeq™, Rotavac^®^, and Rotasiil^®^. The three VP7 epitopes (7-1a, 7-1b, and 7-2) are shown. Amino acids that showed variations between the vaccine strains and the study strains are highlighted in a brown-red color for better visualization.

**Table 3:** Differences in amino acid composition of neutralization epitopes between Rwandan VP4 strains and the VP4 component of the rotavirus vaccine strains.

		Neutralization Epitopes																																				
							8-1						8-2						8-3						8-4					5-1					5-2	5-3	5-4	5-5
	Strain																																					
		100	146	148	150	188	190	192	193	194	195	196	180	183	113	114	115	116	125	131	132	133	135	87	88	89	384	386	388	393	394	398	440	441	434	459	429	306
**Vaccine strains**	**RVA/Vaccine/USA/Rotarix-A41CB052A/1988/G1P[8]**	**D**	**S**	**Q**	**E**	**S**	**T**	**N**	**L**	**N**	**N**	**I**	**T**	**A**	**N**	**P**	**V**	**D**	**S**	**S**	**N**	**D**	**N**	**N**	**T**	**N**	**Y**	**F**	**I**	**W**	**P**	**G**	**R**	**T**	**P**	**E**	**L**	**R**
	**RVA/Vaccine/USA/RotaTeq-WI79-4/1992/G6P[8]**	**D**	**S**	**Q**	**E**	**S**	**T**	**N**	**L**	**N**	**D**	**I**	**T**	**A**	**N**	**P**	**V**	**D**	**N**	**R**	**N**	**D**	**D**	**N**	**T**	**N**	**Y**	**F**	**L**	**W**	**P**	**G**	**R**	**T**	**P**	**E**	**L**	**R**
**Study strains**	**RVA/Human-wt/RWA/UFS-NGS-MRC-DPRU441/2012/G8P[4]**	**D**	**S**	**Q**	**E**	**S**	**T**	**D**	**L**	**N**	**N**	**I**	**T**	**A**	**S**	**Q**	**T**	**N**	**N**	**E**	**N**	**N**	**D**	**N**	**T**	**D**	**Y**	**F**	**L**	**W**	**P**	**G**	**R**	**T**	**P**	**E**	**L**	**R**
	**RVA/Human-wt/RWA/UFS-NGS-MRC-DPRU478/2013/G8P[4]**	**D**	**S**	**Q**	**E**	**S**	**T**	**D**	**L**	**N**	**N**	**I**	**T**	**A**	**S**	**Q**	**T**	**N**	**N**	**E**	**N**	**N**	**D**	**N**	**T**	**D**	**Y**	**F**	**L**	**W**	**P**	**G**	**R**	**T**	**P**	**E**	**L**	**R**
	**RVA/Human-wt/RWA/UFS-NGS-MRC-DPRU582/2013/G8P[4]**	**D**	**S**	**Q**	**E**	**S**	**T**	**D**	**L**	**N**	**N**	**I**	**T**	**A**	**S**	**Q**	**T**	**N**	**N**	**E**	**N**	**N**	**D**	**N**	**T**	**D**	**Y**	**F**	**L**	**W**	**P**	**G**	**R**	**T**	**P**	**E**	**L**	**R**
	**RVA/Human-wt/RWA/UFS-NGS-MRC-DPRU589/2013/G8P[4]**	**D**	**S**	**Q**	**E**	**S**	**T**	**D**	**L**	**N**	**N**	**I**	**T**	**A**	**S**	**Q**	**T**	**N**	**N**	**E**	**N**	**N**	**D**	**N**	**T**	**D**	**Y**	**F**	**L**	**W**	**P**	**G**	**R**	**T**	**P**	**E**	**L**	**R**
	**RVA/Human-wt/RWA/UFS-NGS-MRC-DPRU590/2013/G8P[4]**	**D**	**S**	**Q**	**E**	**S**	**T**	**D**	**L**	**N**	**N**	**I**	**T**	**A**	**S**	**Q**	**T**	**N**	**N**	**E**	**N**	**N**	**D**	**N**	**T**	**D**	**Y**	**F**	**L**	**W**	**P**	**G**	**R**	**T**	**P**	**E**	**L**	**R**
	**RVA/Human-wt/RWA/UFS-NGS-MRC-DPRU596/2013/G8P[4]**	**D**	**S**	**Q**	**E**	**S**	**T**	**D**	**L**	**N**	**N**	**I**	**T**	**A**	**S**	**Q**	**T**	**N**	**N**	**E**	**N**	**N**	**D**	**N**	**T**	**D**	**Y**	**F**	**L**	**W**	**P**	**G**	**R**	**T**	**P**	**E**	**L**	**R**
	**RVA/Human-wt/RWA/UFS-NGS-MRC-DPRU599/2013/G8P[4]**	**D**	**S**	**Q**	**E**	**S**	**T**	**D**	**L**	**N**	**N**	**I**	**T**	**A**	**S**	**Q**	**T**	**N**	**N**	**E**	**N**	**N**	**D**	**N**	**T**	**D**	**Y**	**F**	**L**	**W**	**P**	**G**	**R**	**T**	**P**	**E**	**L**	**R**
	**RVA/Human-wt/RWA/UFS-NGS-MRC-DPRU602/2013/G8P[4]**	**D**	**S**	**Q**	**E**	**S**	**T**	**D**	**L**	**N**	**N**	**I**	**T**	**A**	**S**	**Q**	**T**	**N**	**N**	**E**	**N**	**N**	**D**	**N**	**T**	**D**	**Y**	**F**	**L**	**W**	**P**	**G**	**R**	**T**	**P**	**E**	**L**	**R**
	**RVA/Human-wt/RWA/UFS-NGS-MRC-DPRU607/2013/G8P[4]**	**D**	**S**	**Q**	**E**	**S**	**T**	**D**	**L**	**N**	**N**	**I**	**T**	**A**	**S**	**Q**	**T**	**N**	**N**	**E**	**N**	**N**	**D**	**N**	**T**	**D**	**Y**	**F**	**L**	**W**	**P**	**G**	**R**	**T**	**P**	**E**	**L**	**R**
	**RVA/Human-wt/RWA/UFS-NGS-MRC-DPRU620/2013/G8P[4]**	**D**	**S**	**Q**	**E**	**S**	**T**	**D**	**L**	**N**	**N**	**I**	**T**	**A**	**S**	**Q**	**T**	**N**	**N**	**E**	**N**	**N**	**D**	**N**	**T**	**D**	**Y**	**F**	**L**	**W**	**P**	**G**	**R**	**T**	**P**	**E**	**L**	**R**
	**RVA/Human-wt/RWA/UFS-NGS-MRC-DPRU632/2013/G8P[4]**	**D**	**S**	**Q**	**E**	**S**	**T**	**D**	**L**	**N**	**N**	**I**	**T**	**A**	**S**	**Q**	**T**	**N**	**N**	**E**	**N**	**N**	**D**	**N**	**T**	**D**	**Y**	**F**	**L**	**W**	**P**	**G**	**R**	**T**	**P**	**E**	**L**	**R**
	**RVA/Human-wt/RWA/UFS-NGS-MRC-DPRU637/2013/G8P[4]**	**D**	**S**	**Q**	**E**	**S**	**T**	**D**	**L**	**N**	**N**	**I**	**T**	**A**	**S**	**Q**	**T**	**N**	**N**	**E**	**N**	**N**	**D**	**N**	**T**	**D**	**Y**	**F**	**L**	**W**	**P**	**G**	**R**	**T**	**P**	**E**	**L**	**R**
	**RVA/Human-wt/RWA/UFS-NGS-MRC-DPRU642/2013/G8P[4]**	**D**	**S**	**Q**	**E**	**S**	**T**	**D**	**L**	**N**	**N**	**I**	**T**	**A**	**S**	**Q**	**T**	**N**	**N**	**E**	**N**	**N**	**D**	**N**	**T**	**D**	**Y**	**F**	**L**	**W**	**P**	**G**	**R**	**T**	**P**	**E**	**L**	**R**
	**RVA/Human-wt/RWA/UFS-NGS-MRC-DPRU652/2013/G8P[4]**	**D**	**S**	**Q**	**E**	**S**	**T**	**D**	**L**	**N**	**N**	**I**	**T**	**A**	**S**	**Q**	**T**	**N**	**N**	**E**	**N**	**N**	**D**	**N**	**T**	**D**	**Y**	**F**	**L**	**W**	**P**	**G**	**R**	**T**	**P**	**E**	**L**	**R**
	**RVA/Human-wt/RWA/UFS-NGS-MRC-DPRU653/2013/G8P[4]**	**D**	**S**	**Q**	**E**	**S**	**T**	**D**	**L**	**N**	**N**	**I**	**T**	**A**	**S**	**Q**	**T**	**N**	**N**	**E**	**N**	**N**	**D**	**N**	**T**	**D**	**Y**	**F**	**L**	**W**	**P**	**G**	**R**	**T**	**P**	**E**	**L**	**R**
	**RVA/Human-wt/RWA/UFS-NGS-MRC-DPRU656/2013/G8P[4]**	**D**	**S**	**Q**	**E**	**S**	**T**	**D**	**L**	**N**	**N**	**I**	**T**	**A**	**S**	**Q**	**T**	**N**	**N**	**E**	**N**	**N**	**D**	**N**	**T**	**D**	**Y**	**F**	**L**	**W**	**P**	**G**	**R**	**T**	**P**	**E**	**L**	**R**
	**RVA/Human-wt/RWA/UFS-NGS-MRC-DPRU661/2013/G8P[4]**	**D**	**S**	**Q**	**E**	**S**	**T**	**D**	**L**	**N**	**N**	**I**	**T**	**A**	**S**	**Q**	**T**	**N**	**N**	**E**	**N**	**N**	**D**	**N**	**T**	**D**	**Y**	**F**	**L**	**W**	**P**	**G**	**R**	**T**	**P**	**E**	**L**	**R**
	**RVA/Human-wt/RWA/UFS-NGS-MRC-DPRU666/2013/G8P[4]**	**D**	**S**	**Q**	**E**	**S**	**T**	**D**	**L**	**N**	**N**	**I**	**T**	**A**	**S**	**Q**	**T**	**N**	**N**	**E**	**N**	**N**	**D**	**N**	**T**	**D**	**Y**	**F**	**L**	**W**	**P**	**G**	**R**	**T**	**P**	**E**	**L**	**R**
	**RVA/Human-wt/RWA/UFS-NGS-MRC-DPRU667/2013/G8P[4]**	**D**	**S**	**Q**	**E**	**S**	**T**	**D**	**L**	**N**	**N**	**I**	**T**	**A**	**S**	**Q**	**T**	**N**	**N**	**E**	**N**	**N**	**D**	**N**	**T**	**D**	**Y**	**F**	**L**	**W**	**P**	**G**	**R**	**T**	**P**	**E**	**L**	**R**
	**RVA/Human-wt/RWA/UFS-NGS-MRC-DPRU737/2013/G8P[4]**	**D**	**S**	**Q**	**E**	**S**	**T**	**D**	**L**	**N**	**N**	**I**	**T**	**A**	**S**	**Q**	**T**	**N**	**N**	**E**	**N**	**N**	**D**	**N**	**T**	**D**	**Y**	**F**	**L**	**W**	**P**	**G**	**R**	**T**	**P**	**E**	**L**	**R**
	**RVA/Human-wt/RWA/UFS-NGS-MRC-DPRU7997/2015/G8P[4]**	**D**	**S**	**Q**	**E**	**S**	**T**	**D**	**L**	**N**	**N**	**I**	**T**	**A**	**S**	**Q**	**T**	**N**	**N**	**E**	**N**	**N**	**D**	**N**	**T**	**D**	**Y**	**F**	**L**	**W**	**P**	**G**	**R**	**T**	**P**	**E**	**L**	**R**

Alignment of antigenic residues in VP4 between the wild-type P[4] strains and the strains present in Rotarix^®^ and RotaTeq™. The antigenic residues are categorized into eight epitopes (8-1, 8-2, 8-3, 8-4, 5-1, 5-2, 5-3 and 5-4). Amino acids that display variations between the vaccine strains and the study strains are highlighted in a brown-red color. Those that differ only from RotaTeq™ are highlighted in blue, while those that differ only from Rotarix^®^ are highlighted in green for better visualization.

**Table 4:** Comparison of nucleotide similarity between Rwandan G8P[4] study strains and the closest GenBank strains for different genome segments of group A rotavirus.

Genome Segment	Lineage	Closest Strain from GenBank	Nucleotide Similarity Range (%)	Country
VP7	V	LC177390-RVA/Human-wt/KEN/KDH64/2011/G8P[4]	99.7–100	Kenya
VP4	II	MZ097182-RVA/Human-wt/KEN/KLF1055/2012/G8P[4]	98.8–100	Kenya
VP6	V	KX655466-RVA/Human-wt/UGA/MUL-13–496/2013/G8P[4]	99.5–99.9	Uganda
VP1	V	MZ094284-RVA/Human-wt/KEN/KLF0613/2012/G8P[4]	99.6–99.9	Kenya
	XII	RVA/Vaccine/USA/RotaTeq-BrB-9/1996/G4P[5]	99.9–100	USA
	XII	RVA/Cow-wt/ZAF/Bov1/2009/G6P[5]	97.5–97.6	South Africa
VP2	IV	KX655463-RVA/Human-wt/UGA/MUL-13-496/2013/G8P[4]	99.5–99.9	Uganda
	XIII	HM627543-RVA/Human-wt/KEN/B12/1987/G8P[1]	96.3–96.4	Kenya
VP3	V	KX655464-RVA/Human-wt/UGA/MUL-13-496/2013/G8P[4]	99.3–99.9	Uganda
	X	GU565043-RVA/Vaccine/USA/RotaTeq-WI79-4/1992/G6P[8]	99.6–99.9	USA
NSP1	IV	KX655468-RVA/Human-wt/UGA/MUL-13-496/2013/G8P[4]	99.3–99.9	Uganda
NSP2	V	KX655469-RVA/Human-wt/UGA/MUL-13-496/2013/G8P[4]	99.6–100	Uganda
NSP3	V	KX655503-RVA/Human-wt/UGA/MUL-13-160/2013/G8P[4];	99.4–100	Uganda
NSP4	XV	KX655471-RVA/Human-wt/UGA/MUL-13-496/2013/G8P[4]	99.2–100	Uganda
	XXIII	MT234349- RVA/Buffalo-wt/ZAF/1442/2007/G10P[11]	97.5–97.7	South Africa
NSP5	IV	LC406840- RVA/Human-wt/KEN/KDH1629/2013/G8P[4]	99.5–100	Kenya

Lineage of studied strains, closest strain from GenBank, nucleotide similarity range (%), and country for the 11 genome segments of group A rotavirus.

## Data Availability

All the gene sequences in this study were submitted to the *NCBI GenBank* database under accession numbers OQ201345-OQ201575, and are included in Supplementary Data S1.

## Supplementary Materials

The following supporting information can be downloaded at: https://www.mdpi.com/article/10.3390/pathogens12050658/s1, Supplementary Table S1: Accession numbers of the sequences generated in this study of Rwandan G8P[4] strains; Supplementary Table S2: Accession numbers for the reference sequences used in the construction of the maximum likelihood phylogenetic trees; Supplementary Table S3: The complete nucleotide and amino acid lengths of Rwandan G8P[4] genomic segments; Supplementary Table S4: Nucleotide similarity analysis of Rwandan G8P[4] strains with the respective global GenBank strains.
