# Supplementary material for: The Evolution of Post-Vaccine G8P[4] Group a Rotavirus Strains in Rwanda; Notable Variance at the Neutralization Epitope Sites
Source: Pathogens. 2023 Apr 28;12(5):658. doi: 10.3390/pathogens12050658 (PMC10223037; doi:10.3390/pathogens12050658)
Supplement: Supplementary file 1 [file pathogens-12-00658-s001.zip › Supplementary Table S3.pdf]

Supplementary Table S3

Table S3: The complete nucleotide and amino acid lengths of Rwandan G8P[4] genomic segments

**Genome segments**	**VP1**	**VP2**	**VP3**	**VP4**	**VP6**	**VP7**	**NSP1**	**NSP2**	**NSP3**	**NSP4**	**NSP5**
**Nucleotide length**	3264	2637	2505	2325	1191	978	1458	951	930	525	600
**Amino acid length**	1088	879	835	775	397	326	486	317	310	175	200
