# Supplementary material for: The Evolution of Post-Vaccine G8P[4] Group a Rotavirus Strains in Rwanda; Notable Variance at the Neutralization Epitope Sites
Source: Pathogens. 2023 Apr 28;12(5):658. doi: 10.3390/pathogens12050658 (PMC10223037; doi:10.3390/pathogens12050658)
Supplement: Supplementary file 1 [file pathogens-12-00658-s001.zip › Supplementary Table S2.pdf]

Supplementary Table S2: Accession numbers for the reference sequences used in the construction of the Maximum Likelihood phylogenetic trees

VP7	VP4	VP6	VP1	VP2	VP3	NSP1	NSP2	NSP3	NSP4	NSP5
Outgroup	**Outgroup**	**Outgroup**	**Outgroup**	**Outgroup**	**Outgroup**	**Outgroup**	**Outgroup**	**Outgroup**	**Outgroup**	**Outgroup**
**GU723327**	JX406750	JX406752	JX406747	JX406748	JX406749	JX406751	JX406754	JX406753	JX406756	JX406757
Lineage I	**Lineage I**	**Lineage I**	**Lineage I**	**Lineage I**	**Lineage I**	**Lineage I**	**Lineage I**	**Lineage I**	**Lineage I**	**Lineage I**
**GQ41454**<br>**KC870012**<br>**KC870004**<br>**JN71110**<br>**JN71112**	LC001901<br>KC443590<br>HQ650119	DQ870507<br>HQ650121<br>KC443591	DQ870505<br>KC443587	HQ650117	HQ650118<br>KC443589	HQ650120<br>KC443582	HQ650123	HQ650122<br>KC443584	HQ650125	HQ650126<br>KC443586
Lineage II	**Lineage II**	**Lineage II**	**Lineage II**	**Lineage II**	**Lineage II**	**Lineage II**	**Lineage II**	**Lineage II**	**Lineage II**	**Lineage II**
**FJ347105**<br>**KF500212**<br>**MF940553**<br>**MF940609**<br>**MF940611**	KJ752665<br>KJ752461<br>KF636314<br>MG892018<br>MG926728	JF304930	DQ146693<br>JF304915	JF304916<br>JF304927	EF583035<br>JF304928	AB975785<br>GQ414546	EU708908<br>EU708930<br>EU708952	JF304934<br>EF672593	DQ525190<br>KC178746	KC178755<br>JQ715677
Lineage III	**Lineage III**	**Lineage III**	**Lineage III**	**Lineage III**	**Lineage III**	**Lineage III**	**Lineage III**	**Lineage III**	**Lineage III**	**Lineage III**
**FJ611782**<br>**AF20706**<br>**AF034852**<br>**EF672560**	AB733131	AB733132	LC438390<br>AB733133	AB733134<br>LC438391	LC438392<br>AB733135	AB733136<br>LC438396	AB733137<br>LC438397	AB733138	D88829<br>LC438399	AB091727<br>LC438400
Lineage IV	**Lineage IV**	**Lineage IV**	**Lineage IV**	**Lineage IV**	**Lineage IV**	**Lineage IV**	**Lineage IV**	**Lineage IV**	**Lineage IV**	**Lineage IV**
**AF104104**<br>**JX156636**<br>**FJ861661**	MG181837<br>MG652350<br>JQ069668<br>KP882275<br>KC443159<br>KC443258<br>KP007171<br>KP753181	AB762771<br>AY787645	AB762772<br>AY787653	KC443785<br>EF554083<br>AY787652<br>LC065021	AB762774<br>AY787654	KJ751619<br>EF554088<br>AB762775<br>MG181827<br>JX965145<br>MT767401<br>KC442887<br>KU248378<br>KJ753518	AY787648	LC002076<br>AB762777	AB762778<br>AY87650	LC065027<br>AB971565<br>MH301253<br>KC443783<br>JX965157<br>KF716441<br>KU248393
Lineage V		**Lineage V**	**Lineage V**	**Lineage V**	**Lineage V**	**Lineage V**	**Lineage V**	**Lineage V**	**Lineage V**	
**KJ751607**<br>**LC177390**<br>**MG891987**<br>**KX655500**<br>**KF636316**		EF554086	KU059766<br>KU550262	EU708891<br>EU708902	LC026108<br>KC443786<br>JX965139<br>KC443786<br>KC443622<br>KF716382	JX416214<br>EF672592	KU059773<br>LC002074.1<br>KC442959<br>KR705329<br>MN223989<br>JX965148	LC074739<br>EF554090<br>MG181928<br>KJ752125<br>KC571499<br>KU361002	HM467979<br>HQ641371	

					KF716381<br>LC060819<br>KR705214<br>LC026108<br>KR705401<br>MG181768<br>KJ752996		KU059773<br>KJ753821<br>LC002074<br>KJ753486<br>KM660141<br>KJ583192	KP882535<br>KP188805<br>MF184795<br>KJ752615		
		**Lineage VI**	**Lineage VI**	**Lineage VI**	**Lineage VI**			**Lineage VI**	**Lineage VI**	
		KJ748480<br>KP882628	EF554082<br>KJ751624	KP882630<br>KP882685	JX416213<br>KP882477			JX416216<br>AB753500	AB796456<br>LC228324	
		**Lineage VII**	**Lineage VII**	**Lineage VII**	**Lineage VII**			**Lineage VII**	**Lineage VII**	
		LC095956<br>LC169856	FJ031024<br>JQ013502	FN665689<br>GU384191	GU937879<br>LC105582			EF672558<br>JQ863317	KX265688<br>AB975935	
		**Lineage VIII**	**Lineage VIII**	**Lineage VIII**	**Lineage VIII**				**Lineage VIII**	
		EF583020	LC065020<br>FN665688	HQ834198<br>JQ013503	EF554106				AB938299<br>GU937886	
		**Lineage IX**	**Lineage IX**	**Lineage IX**	**Lineage IX**				**Lineage IX**	
		KP882925<br>EF554152	EF583017<br>EF576937	GU827407<br>EF554105	JQ013504<br>HQ834207				LC119113<br>KP882481	
		**Lineage X**	**Lineage X**	**Lineage X**	**Lineage X**				**Lineage X**	
		DQ870496<br>EF554130	GU296420<br>EF554104	AB748581<br>KC815659	EF583015<br>DQ70495				KM660203<br>KM660192	
		**Lineage XI**	**Lineage XI**	**Lineage XI**	**Lineage XI**				**Lineage XI**	
		EF583016<br>KC175098	LCO7473<br>LC102997	EF583014<br>JF421976	EF554084<br>GU296424				KUO59775	
		Lineage XII	Lineage XII	Lineage XII	Lineage XII				Lineage XII	
		AY740737<br>KP258402	KJ919361<br>KC175269	JX271002<br>EF583042	JF304917<br>AB751564				LC074740<br>LC102992	
		Lineage XIII	Lineage XIII	Lineage XIII	Lineage XIII				Lineage XIII	
		AB573073<br>DQ838647	JX271001<br>JF693037	KM454482<br>KM454493	KJ748478<br>KJ748467				EF592592	
		Lineage XIV	Lineage XIV	Lineage XIV					Lineage XIV	
		EF554108<br>GU827410	JN903527<br>JQ345489	EF554127<br>KF636257					JF304924<br>AB755562	
		Lineage XV							Lineage XV	
		JQ345494<br>AB908927							GU25961<br>DQ005105	
									Lineage XVI	
									EF554135	
									Lineage XVII	
									GU827415<br>GU296416	

									Lineage XVIII	
									KY426806<br>JN831207	
									Lineage XIX	
									KC257089<br>KP198645	
									Lineage XX	
									EF554157<br>KJ940160	
									Lineage XXI	
									KJ870898<br>KJ748485	
									Lineage XXII	
									EF672561<br>KP941135	
									Lineage XXIII	
									MG181621<br>MG181929	
									Lineage XXIV	
									JN248454<br>KP752503<br>LC055556	
									Lineage XXV	
									FJ031022<br>JQ031148	
									Lineage XXVI	
									AJ311728<br>AJ311729	
									Lineage XXVII	
									JQ687223<br>JN903510	
									Lineage<br>XXVIII	
									KC815689	
									Lineage XXIX	
									GU181282	
									Lineage XXX	
									KY972050<br>KY972002	
